# Graphene based widely-tunable and singly-polarized pulse generation with random fiber lasers

**DOI:** 10.1038/srep18526

**Published:** 2015-12-21

**Authors:** B. C. Yao, Y. J. Rao, Z. N. Wang, Y. Wu, J. H. Zhou, H. Wu, M. Q. Fan, X. L. Cao, W. L. Zhang, Y. F. Chen, Y. R. Li, D. Churkin, S. Turitsyn, C. W. Wong

**Affiliations:** 1 Key Laboratory of Optical Fiber Sensing and Communications (Education Ministry of China), University of Electronic Science and Technology of China, Chengdu 610054, China; 2 Mesoscopic Optics and Quantum Electronics Laboratory, University of California, Los Angeles, CA90095, United States; 3 State Key Laboratory of Electronic Thin Films and Integrated Devices, University of Electronic Science and Technology of China, Chengdu 610054, China; 4 Aston Institute of Photonic Technologies, Aston University, Birmingham, B47ET, United Kingdom; 5 Laboratory of Nonlinear Photonics, Novosibirsk State University, Novosibirsk, 630090 Russia; 6 Institute of Automation and Electrometry, Siberian Branch of the Russian Academy of Sciences, Novosibirsk, 630090, Russia

## Abstract

Pulse generation often requires a stabilized cavity and its corresponding mode structure for initial phase-locking. Contrastingly, modeless cavity-free random lasers provide new possibilities for high quantum efficiency lasing that could potentially be widely tunable spectrally and temporally. Pulse generation in random lasers, however, has remained elusive since the discovery of modeless gain lasing. Here we report coherent pulse generation with modeless random lasers based on the unique polarization selectivity and broadband saturable absorption of monolayer graphene. Simultaneous temporal compression of cavity-free pulses are observed with such a polarization modulation, along with a broadly-tunable pulsewidth across two orders of magnitude down to 900 ps, a broadly-tunable repetition rate across three orders of magnitude up to 3 MHz, and a singly-polarized pulse train at 41 dB extinction ratio, about an order of magnitude larger than conventional pulsed fiber lasers. Moreover, our graphene-based pulse formation also demonstrates robust pulse-to-pulse stability and wide-wavelength operation due to the cavity-less feature. Such a graphene-based architecture not only provides a tunable pulsed random laser for fiber-optic sensing, speckle-free imaging, and laser-material processing, but also a new way for the non-random CW fiber lasers to generate widely tunable and singly-polarized pulses.

Random lasers have received considerable attention due to the unique properties that distinguish them from conventional resonant cavity lasers, including modeless, cavity-less, low coherence output, simple design, and good reliability operation^1^,^2^,^3^. Recently random fiber lasers based on the Rayleigh scattering and Raman effects in standard optical fibers have been advanced^4^,^5^,^6^. These fiber lasers with high quantum efficiency and large spectral tunability open up a new direction in both science and fiber-optic technologies. Reliable high-power operation, long-distance distributed fiber-optic sensing, and speckle-free imaging are promising novel areas for random fiber laser applications^7^,^8^,^9^,^10^. The development of pulsed random fiber lasers can also open up new applications, including ultrafast optical communications, precision sensing, high-resolution imaging, and next-generation laser processing and medical applications^11^,^12^,^13^. However, since random fiber lasers are modeless, it has been a fundamental scientific challenge to achieve mode-locking or to generate short pulses in such systems. Conventional methods of pulse generation in fiber resonators, such as mode-locking, spatial interference, and *Q*-switching, are generally not applicable to random fiber lasers with random feedbacks^14^,^15^.

Graphene is an attractive two-dimensional material with a variety of exceptional electronic and photonic properties, and has been proposed in various applications^16^,^17^,^18^. Due to its unique band structure, graphene serves as an excellent saturable absorber^19^,^20^,^21^. Accordingly, effective graphene-based mode-locked lasers and all-optical fast modulators have been proposed^22^,^23^,^24^,^25^. As a waveguide, graphene also provides exceptional properties in polarization dependent transmissions^26^,^27^,^28^,^29^, serving as a natural polarizer^30^,^31^,^32^. Moreover, compared to other materials for optical applications, such as carbon nanotubes or cadmium compounds^33^,^34^, graphene has some merits: (1) broadband high nonlinearity with ultrafast response^35^,^36^; (2) high-power sustainability^14^,^20^,^21^,^22^, (3) high mechanical strength and durability^37^, and (4) compatibility with silicon waveguide and fiber hybrids^36^,^38^,^39^. With graphene-based hybrids as a static absorber, mode-locking and *Q*-switching have been examined^31^,^32^, albeit still requiring a conventional resonant cavity lasing structure.

Here we report graphene-based pulse generation with cavity-free random fiber lasers, for the first time. By dynamically modulating the polarization via a polarization rotator (PR) and then transmitting through a designed graphene-coated *D*-shaped fiber (GDF), the continuous wave (CW) from the arbitrarily-polarized random fiber laser is transformed into coherent sub-nanosecond pulses even in the non-resonant single-pass configuration. The PR and the GDF hybrid waveguide serves simultaneously as the linearly polarized pulse generator (polarization modulation) and the pulse reshaper (saturable absorption). The pulses generated from the GDF not only inherit merits of the CW random fiber laser such as a high pump-power Stokes conversion efficiency, but also offer unique advantages such as a power-dependent singly-polarized output with polarization extinction ratios up to 41 dB, and wide tunability in *both* pulsewidths and repetition rates, across two and three orders of magnitude respectively.

## Results

### Architecture of the pulse generator

**Figure 1:**
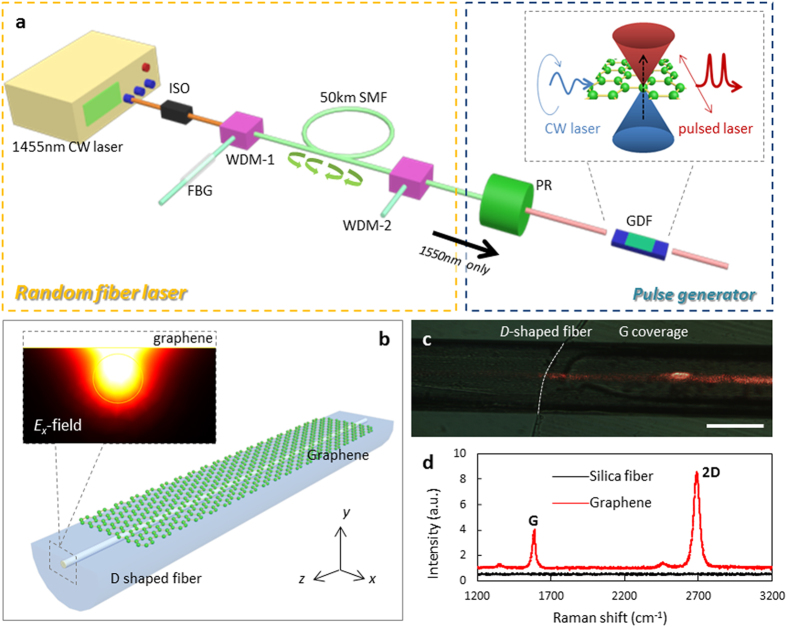
Widely tunable and singly-polarized pulse generation for random fiber lasers. (**a)** Schematic of the short pulse generation. A CW random fiber laser (yellow box) is polarization-rotated by a polarization rotator (PR), followed by intensity modulation with the graphene-coated *D*-shaped fiber (GDF). (**b)** Structure of the graphene-coated *D*-shaped fiber, with a graphene monolayer (green atoms) deposited on the top-side polished surface of a *D-*shaped fiber. The coverage length of the graphene *L*_*G*_ is 16.7 mm and the polished depth of the *D*-shaped fiber *d* is 57 μm. Inset: Simulated *E*_*x*_-field distribution, with the graphene film over cladding. (**c)** Optical micrograph of the graphene-coated *D-*shaped fiber. The boundary of the graphene coverage is illustrated with the white dashed line. By coupling 633 nm light into the fiber, the graphene-enhanced scattering is illustrated in the red scattering regions. Scale bar: 50 μm. (**d)** Raman spectra of the CVD-grown graphene monolayer and the *D*-shaped silica fiber. The weak *D*-peak, the FWHM of the *G*-peak (32.5 cm^−1^) and *2D*-peak (38.2 cm^−1^), and the *G*-to-*2D* peak ratio (0.36) means that the graphene film is of monolayer and uniformity.

Figure 1(a) illustrates the concept of generating highly polarized pulses for a CW random fiber laser. Determined by the graphene’s anisotropy, the loss on the TM-polarization is much higher than that on the TE-polarization^28^,^38^. When light transmits along the GDF, as shown schematically in the inset, it can be regarded as a *x*-polarization transmission filter. With the *x*-polarization periodically modulated by the PR, the transmission is a sinusoidal-like temporal wave after passing the GDF. Simultaneously, with Pauli blocking and self-phase modulation at high input powers^21^,^22^,^40^, the temporal width of sinusoidal-like wave can be dramatically compressed to form narrow pulses. In this process, the polarization rotation rate determines the pulse repetition rate while the saturable absorption efficiency determines the pulsewidth. Theoretical analysis is detailed in Supplementary Information Section S1.

With the finite element method (FEM), an example modeled *E*_*x*_-field distribution is shown in Fig. 1(b) with other polarization and fields shown in Supplementary Information Section S1. For the *D*-shaped fiber without graphene coverage, the *x*-polarized component and the *y*-polarized component have almost equal intensity. In comparison, with graphene cladding, the electromagnetic field distribution is pulled close to the graphene film and the *x*-polarized component is much stronger than the *y*-polarized component. With the graphene-based evanescent field enhancement, the transmitted CW random lasing field interacts slightly with the graphene film (detailed in Supplementary Information Section S1).

### Graphene-coated *D*-shaped fiber

The *D*-shaped fiber was carefully polished with good uniformity and small surface roughness, with an insertion loss less than 10 dB over a polished length of 20 mm. Detailed fabrication process is shown in Supplementary Information Section S2. The fabrication of the GDF with a long length and a highly uniform surface is crucial to ensure effective light-graphene interaction and low scattering losses. Significantly, the polarization dependent loss (PDL) determines the initial polarization extinction ratio (PER) and the peak-to-noise ratio (PNR) of the generated pulses, while the absorption efficiency dominates how strongly the pulses could be modulated optically. Figure 1(c) shows an optical micrograph of the graphene cladded *D*-shaped fiber, under 633 nm transmission for clarity. In order to prepare high-quality graphene film and transfer it to the *D*-shaped fiber properly, monolayer CVD graphene was used^41^ instead of exfoliated graphene. With the wet-transfer technique^36^, we successfully covered graphene over several centimeters of the *D*-shaped fiber surface with good flatness and uniformity, even when the fiber was polished down with 58 μm depth (details in Supplementary Information Section S3). Figure 1(d) shows the Raman spectrum of the GDF, which verifies that the deposited graphene is of sufficient quality for our measurement. For a GDF with 1.67  cm length, the excess graphene loss is measured to be ~22 dB.

### Random fiber lasing

**Figure 2:**
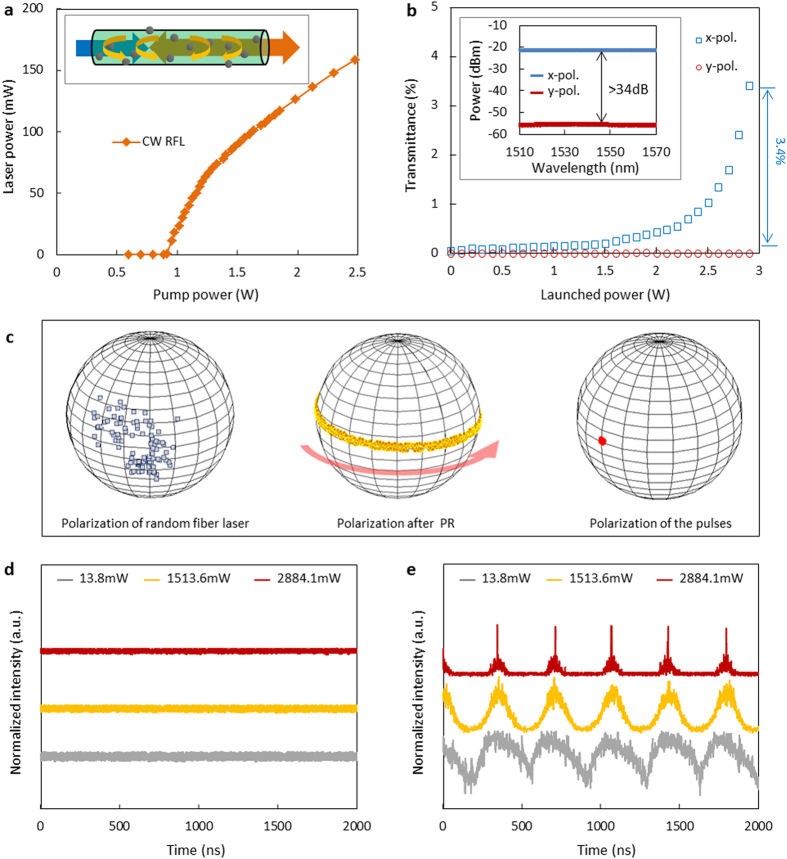
Polarization selection and pulse generation of random fiber lasers. (**a**) Output power of the CW random fiber laser. Inset: Distributed feedback of random fiber laser. (**b)** Polarization dependent transmittance of the graphene-coated *D-*shaped fiber at 1550 nm under CW driving (blue squares: *x-*polarization; red circles: *y-*polarization). Inset: transmission spectra under 9.8 mW (blue curve: *x-*polarization; red curve: *y-*polarization). (**c)** Poincaré map of the CW random fiber laser before the polarization rotator *PR* (low polarization degree), after the *PR* (rotated polarization), and after the graphene-coated *D*-shaped fiber (linear polarization). (**d**,**e**) Intensity dynamics of the random fiber laser measured before and after the graphene-coated *D*-shaped fiber at 13.8 mW (grey curve), 1.51 W (yellow curve), and 2.88 W (red curve), respectively. The pulse repetition period in (**e**) is ~360 ns.

A high-power CW laser at 1455 nm serves as the pump for the random fiber laser. After the accumulation of Rayleigh scatterings and Raman effects in a 50 km single mode fiber (SMF), a CW random fiber laser at 1550 nm is obtained. Random fiber lasers with other wavelengths are also achievable from their stimulated Raman scattering nature^42^. A fiber Bragg grating (FBG) serves as a mirror to reduce the lasing threshold^43^, and a WDM is used to filter off the pump component (detailed in Supplementary Information Section S4). Figure 2(a) shows the dependence of the laser power on the pump power. The lasing threshold is 0.88 W. When the pump power reaches 2.5 W, the output power of the random fiber laser measured after the WDM-2 is 160 mW. Considering the loss of the 50 km long system is over 10 dB, the pump-to-Stokes conversion efficiency of the random fiber laser is estimated conservatively to be above 60%, which could be even higher with short fiber lengths^42^.

### Pulse generation and characteristics

The polarization output of the random fiber laser is next modulated periodically using the PR, with a rotation speed tunable from 1 kHz to 3 MHz, prior to launching into the GDF. The power-dependent polarization selectivity and saturable absorption of the GDF are shown in Fig. 2(b). With a broadband tunable laser with a low average power of 9.8 mW, the GDF has demonstrated its natural PDL as shown in the inset of Fig. 2(b). In the window of 1510 nm to 1570 nm, the transmission in the *x*-polarization was approximately −22 dBm, while the transmission in the *y*-polarization was approximately −57 dBm for a PER in excess of 34 dB. When the launched power of the GDF increases gradually, saturable absorption begins to occur along the GDF. Supplementary Information Section S5 details the saturable absorption and theoretical modeling. When the launched power of the *x-*polarized laser launched in the GDF increased from 1 mW to 2.9 W, the transmittance of the GDF increases from ~0.05% to ~3.4%. Contrastingly, for the *y-*polarized laser with launched power of 2.9 W, the transmission stays predominantly constant with only a 0.0002% increase, within the noise level and negligible. This indicates that much higher power is needed to use the *y-*polarized light to saturate the GDF. Such a polarization-dependent saturable absorption is determined compositely by the polarization dependent transmission of the waveguide, and the polarization dependent nonlinear response of graphene.

The polarization properties of the graphene based random fiber laser are shown in Fig. 2(c), illustrating the measured Stokes vectors before the PR, after the PR, and after the GDF. Details of the polarization selectivity are shown in Supplementary Information Section S6. Initially, the CW random fiber laser has a low polarization degree of less than 30%, with a random polarization state. The PR then polarizes it and rotates its polarization, with subsequent polarized transmission filtering by the GDF.

The intensity dynamics after the PR are measured by a real-time oscilloscope, comparing before and after the GDF. Under 2.8 MHz rotation, Fig. 2(d) shows the transmission with increasing powers up to 2.88 W where a constant CW transmission is observed before the GDF. In contrast, after the GDF, as shown in Fig. 2(e), the CW laser becomes a sinusoidal-like oscillation. With increasing launched powers from 13.8 mW to 2.88 W, the sinusoidal-like oscillation is compressed to be sub-ns pulses gradually, at a constant repetition rate.

**Figure 3:**
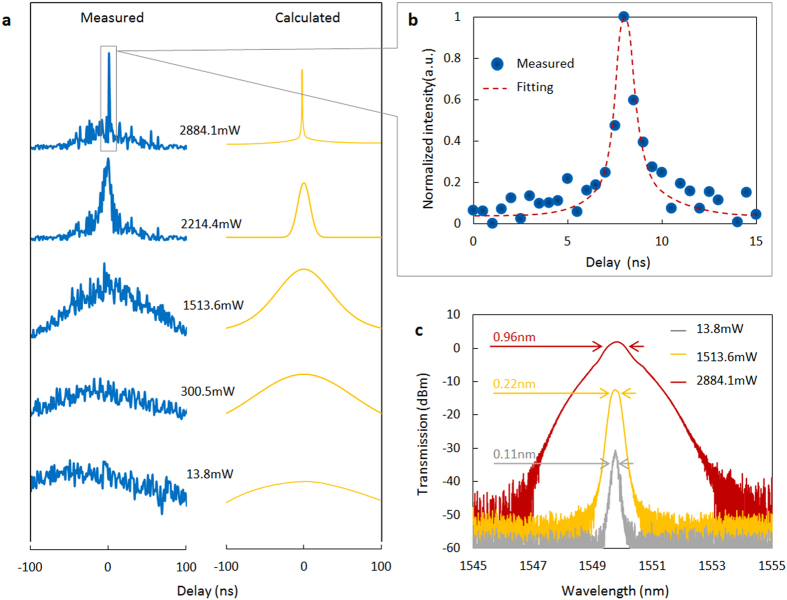
Temporal-spectra structure of the graphene-based pulse generation. **(a)** Measured (left column) and calculated (right column) temporal profiles of the generated pulses at 1549.5 nm and with a 2.8 MHz repetition rate. Different launched powers are examined to illustrate the pulse formation. (**b)** Zoom-in of the pulse at 2.88 W. Raw measurements are illustrated in the blue data points, with a Gaussian fit in the black dashed line. The measured pulsewidth is estimated to be ~900 ps. (**c)** Measured spectra of the pulsed graphene random fiber laser at launched power of 13.8 mW (grey), 1513.6 mW (yellow) and 2884.1 mW (red). The spectral 3-dB width increases from 0.11 nm to 0.96 nm, and correlates with the shortened temporal pulsewidths.

Figure 3(a) shows the progression of the generated pulses under increasing launched powers and a fixed 2.8 MHz repetition rate. The (left column) blue data points are the measured pulsewidths while the (right column) yellow curves are from the theoretical calculations, based on the theoretical analysis in Supplementary Information Section S1. With the launched power increasing from 13.8 mW to 2.88 W, the sinusoidal-like waveform with full width at half maximum (FWHM) more than 180 ns is compressed to be narrow pulses gradually. The pulse generation becomes obvious when the launched power higher than 2.2 W. At the launched power of 2.88 W, the duty cycle is lower than 0.3%, where the duty cycle means the value of the FWHM of a single pulse comparing the temporal period of the PR. Figure 3(b) demonstrates the ~900 ps pulse in detail. With the high power induced saturable absorption, the pulses are generated by compressing the sinusoidal-like waveform more than two orders of magnitude.

The correspondingly pulse spectra is shown in Fig. 3(c). At higher pulse energies based on the higher launched power, chirp and self-phase modulation is evident. At 13.4 mW initially, the 3-dB full-width half-maximum of the random fiber laser spectrum is ~0.1 nm, since the FBG performs as a narrow filter. At 2.88 W, the 3-dB spectral width increases to 0.96 nm and is dominated by the graphene pulse reshaping. Supplementary Information Section S7 also notes the 30-dB spectral width to illustrate the self-phase-modulation and chirp effects. Here the pulse compression and spectra broadening is modeled via the beam analysis and Fourier transform methods. The GDF formed pulses and details of the numerical modeling are illustrated in Supplementary Information Section S1.

### Tunability

**Figure 4:**
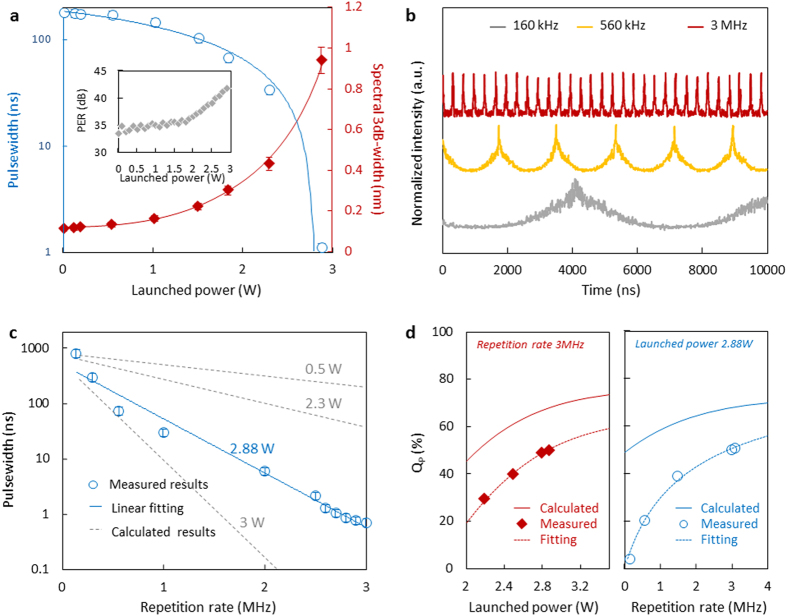
Characteristics and tunability of the generated pulses. **(a)** Pulsewidths (blue circles) and spectra width (red diamonds) for increasing launched power at 1549.5 nm. Inset: Power-dependent polarization extinction ratio at 1549.5 nm. (**b)** Intensity dynamics at 160 kHz, 560 kHz, and 3 MHz repetition rates at 2.88 W and 1549.5 nm. (**c)** Pulsewidth dependence on repetition rate, with blue circles representing the measurement data including error bars. The grey dashed lines are the theoretical models for different launched powers. (**d)** Pulse quality dependence on launched power (Left side, with a fixed repetition rate 3 MHz), and pulse quality dependence on repetition rate (right side, with a fixed launched power 2.88 W).

Figure 4(a) shows the dependence of the compressed-width and spectra for increasing the launched power of the GDF and at a fixed repetition rate, tuning over two orders of magnitude in the pulsewidth up to 2.88 W, with a fixed repetition of 2.8 MHz. With launched powers in excess of 2.88 W, we note that thermal damage of graphene starts to appear, limiting further compression currently. Moreover, considering the polarization-dependent saturable absorption of the GDF, the PER of the pulses is also power-controllable, as shown in inset of Fig. 1(a). With the launched power increasing from 1 mW to 2.88 W, the PER of the pulses increases gradually, due to stronger saturation in *x-*polarization. The PER reaches ~41 dB at ~2.88 W, one of the highest values observed for high power polarizers.

Figure 4(b) shows the tuning of the repetition rate through the PR rotation rate, for a fixed launched power at 2.88 W, illustrated at 160 kHz, 560 kHz, and 3 MHz, for example. By increasing repetition rate, the sinusoidal-like pedestal is suppressed dramatically, and the pulse quality increases to 50.9%, at the launched power of 2.88 W and the repetition rate of 3 MHz. The time jitter of the pulse train generated by the PR and the GDF is mainly determined by the stability of the PR, whose temporal instability is lower than 0.1%. Hence, a higher repetition rate results in a lower time jitter.

Figure 4(c) shows the pulsewidths versus the repetition rates, starting from 1 kHz to 3 MHz and with the pulsewidth depending exponentially on the repetition rate. A higher repetition rate also brings about narrower pulsewidth, as the pulses are compressed from the initial sinusoidal-like envelope formed by the GDF, based on its polarization selectivity. When the repetition rate increases, the width of the initial sinusoidal-like peaks becomes narrower. Hence, after compression, these faster sinusoidal-like peaks are transformed into even narrower pulses. Based on the numerical scaling, we note that the pulsewidth could reach sub-picosecond levels at a repetition rate above 6 MHz.

In addition, as the pulses are gradually compressed from the initial sinusoidal-like wave, a weak sinusoidal-like envelope is always persistent at the pulse pedestal, distinct from conventional cavity solitons or cavity mode-locking. Such a residual pedestal would deteriorate the pulse quality, which could be described as *Q*_*P*_* *=* E*_*FWHM*_*/E*_*0*_, here *E*_*FWHM*_ is the energy in the full width at half maximum (FWHM) of the pulse, *E*_*0*_ is the energy of the pulse integrated over its period^44^. However, by increasing launched power or repetition rate, the sinusoidal-like pedestal could be dramatically suppressed, so that the pulse quality could be improved, as shown in Fig. 4(d). Referring Supplementary S1, calculated curve suggests that the compressed pulses can have a *Q*_*P*_ > 70%. However, limited by the background noise and instability of the PR, the measured pulse quality is relatively lower. In the experiment, the maximum measured *Q*_*P*_ is 50.9%, with launched power 2.88 W and repetition rate 3 MHz.

## Discussion

Distinct from conventional mode-locking lasers, pulse generated by the single-pass GDF configuration is compressed from sinusoidal-like envelope. To achieve short pulsewidth and acceptable pulse quality, relatively high launched power and fast PR is necessary. However, to generate pulses with cavity-free random fiber lasers, external methods are necessary. Fortunately, the GDF based pulse generation has exceptional properties, and could be further improved in the future, by optimizing the smoothness/uniformity of the GDF, the performance of the PR, and increasing the launched power or the modulation speed of polarization.

In addition, beyond the random fiber lasers, the graphene based pulse generator could also be widely applied on typical non-random CW lasers in common fiber systems, as the polarization selectivity and the saturable absorption of the GDF is unique and universal. Hence, it provides a new way for the non-random CW lasers to generate widely tunable and singly-polarized pulses.

In summary, we have demonstrated a graphene based 900 ps and widely-tunable pulse generation, with up to 41 dB polarization extinction for random fiber lasers. The single-pass configuration affords unprecedented widely-tunable pulses over two orders of magnitude in pulsewidth and three orders of magnitude in repetition rate, drawing from the synergistic advantages of graphene photonics and random fiber lasers. This not only inspires exploration of the full potential of graphene-based fiber devices for ultrafast photonics, but also the realization of pulsed random fiber lasers towards sensing, imaging and laser-material processing applications.

## Methods

### Theoretical analysis and numerical simulations

The electric field distribution of the graphene-coated *D-*shaped fiber (GDF), the polarization-dependent transmission, and fast pulse generation were theoretically investigated and numerically simulated. To estimate the power density in the GDF, the field distribution of the graphene-coated *D-*shaped fiber was simulated by *COMSOL* (considering the index of graphene to be determined by its conductivity) by applying *ε*_*g,eq*_* = −σ*_*g,i*_*/ω*Δ* + iσ*_*g,r*_*/ω*Δ and *ω*^*2*^*μ*_*0*_*ε = n*^*2*^*k*_*0*_^*2*^; see Supplementary S1.1. Moreover, modulated by the graphene-coated *D*-shaped fiber, the CW light from the random fiber laser would be transformed into highly polarized pulses with power that varies with time.

### GDF fabrication and characterization

The GDF samples were fabricated by following steps. Firstly, graphene was grown by using the CVD method on Cu foil. Secondly, after removing Cu by FeCl_3_ solution, a soft and flexible PMMA/graphene was prepared. Thirdly, the PMMA/graphene hybrid was covered on the polished surface of a *D-*shaped fiber, which was carefully fabricated with the polished depth of 58 μm and polished length ~20 mm. Finally, the PMMA was removed by acetone vapor. The *D*-shaped fiber and graphene were clearly identified and characterized using optical microscopes (OPM), scanning electronic microscopes (SEM), and a Raman spectrum analyzer. More details are shown in Figs S2 and S3.

### Measurement and simulation of the polarization dependent saturable absorption

A polarization controller (PC) is used to adjust and fix the input polarization. The power launched into the graphene-coated *D-*shaped fiber can reach 3.5 W. The power meter with resolution of 0.1 dBm was used to detect the output power at 1550 nm. The transmittance could be calculated as *P*_*B*_*/P*_*A*_. Figure S5 in Supplementary Information shows the method and the results.

### GDF based polarization selectivity measurement

To verify the broadband polarization selectivity of the GDF, an experimental setup was built as shown in Supplementary Fig. S6. Light from 1510 nm to ~1570 nm was launched from a tunable fiber laser (81960A, Agilent, USA), and measured by a high resolution OSA (8163B, Agilent, USA) and a power meter. The output power of the laser is fixed at 9.8 mW. A polarizer was used to control the launched polarizations.

## Additional Information

**How to cite this article**: Yao, B. C. *et al.* Graphene based widely-tunable and singly-polarized pulse generation with random fiber lasers. *Sci. Rep.* **5**, 18526; doi: 10.1038/srep18526 (2015).

## Supplementary Material

Supplementary Information
