# Supplementary material for: Graphene based widely-tunable and singly-polarized pulse generation with random fiber lasers
Source: Sci Rep. 2015 Dec 21;5:18526. doi: 10.1038/srep18526 (PMC4685245; doi:10.1038/srep18526)
Supplement: Supplementary Information [file srep18526-s1.pdf]

Supplementary Information

Graphene based widely-tunable and singly-polarized pulse generation with random fiber lasers

B. C. Yao[1,2], Y. J. Rao[1*], Z. N. Wang[1], Y. Wu[1], J. H. Zhou[3], H. Wu[1], M. Q. Fan[1], X. L. Cao[1], W. L. Zhang[1], Y. F. Chen[3], Y. R. Li[3], D. Churkin[4,5,6], S. Turitsyn[4,5], and C. W. Wong[2]

[1] *Key Laboratory of Optical Fiber Sensing and Communications (Education Ministry of China), University of Electronic Science and Technology of China, Chengdu 610054, China*

[2]*Mesoscopic Optics and Quantum Electronics Laboratory, University of California, Los Angeles, CA 90095, United States*

[3]*State Key Laboratory of Electronic Thin Films and Integrated Devices, University of Electronic Science and Technology of China, Chengdu 610054, China*

[4]*Aston Institute of Photonic Technologies, Aston University, Birmingham, B47ET, United Kingdom*

[5] *Laboratory of Nonlinear Photonics, Novosibirsk State University, Novosibirsk, 630090 Russia*

[6]*Institute of Automation and Electrometry, Siberian Branch of the Russian Academy of Sciences, Novosibirsk, 630090, Russia*

[*] Corresponding author: yjrao@uestc.edu.cn

S1. Theoretical analysis and numerical simulations.

1.1 Electric field distributions of the graphene-coated *D*-shaped fiber.

According to the waveguide theory, the spatial distribution of mode field of the graphene-coated *D*-shaped fiber (GDF) is described in Eq. (S1) and Eq. (S2), which is determined by the effective refractive index of the GDF. Here f_x and f_y are the *x*- and *y*-polarized component of field intensity, $n_{eff}k_0$ is the propagation constant, ω is the photonic frequency, $\mu_0=4\pi\times10^{-7}$H/m, σ_g is the conductivity of the graphene, $f_d(\varepsilon) =\{exp[(\varepsilon-\mu)/k_BT]+1\}^{-1}$ is the Fermi-Dirac distribution, k_B is the Boltzmann's constant, *j* is the imaginary unit and *e* is the unit charge [S1-S3]. The distribution of the n_{eff} of a graphene-coated *D*-shaped fiber is a function of both σ_g and *D* (the polishing depth), which has been investigated in Ref. [S4-S5].

Accordingly, zoom-in images of the electric fields are shown in Fig. S1(a). The permittivity of the graphene is related to its conductivityσ_g as $\varepsilon_{g,eq}$= -$\sigma_{g,i}/\omega\triangle$ +$i\sigma_{g,r}/\omega\triangle$, where $\triangle$=0.4 nmis the thickness of graphene. Hence, its index was calculated by applying $\omega^2\mu_0\varepsilon=n^2k_0^2$ [S5].

$$\begin{bmatrix} \frac{\partial^2 f_y}{\partial x \partial y} - \frac{\partial^2 f_x}{\partial y^2} \\ \frac{\partial^2 f_x}{\partial x \partial y} - \frac{\partial^2 f_y}{\partial x^2} \end{bmatrix} = (i\omega\mu_0\sigma_g + k_0{}^2 n_{eff}{}^2) \begin{bmatrix} f_x \\ f_y \end{bmatrix} \quad \text{(S1)}$$

$$\sigma_g = \frac{je^2(\omega - j/\tau)}{\pi\hbar^2} \left\{ \frac{1}{(\omega + j/\tau)^2} \int_0^\infty \varepsilon \left[\frac{\partial f_d(\varepsilon)}{\partial \varepsilon} - \frac{\partial f_d(-\varepsilon)}{\partial \varepsilon} \right] d\varepsilon - \int_0^\infty \left[\frac{f_d(-\varepsilon) - f_d(\varepsilon)}{(\omega + j/\tau)^2 - 4(\varepsilon/\hbar)^2} \right] d\varepsilon \right\} \quad \text{(S2)}$$

1.2 Graphene based highly polarized pulse generation and narrowing.

Considering the frequency of the polarization rotator (~MHz level) is orders slower than the frequency of the transmitting light (~200 THz), the average intensity of the CW random fiber laser in front of the GDF is regarded as a constant independent from time, the E_I. Rotated by the polarization rotator periodically, the components in *x*- and *y*-polarization before launched into the GDF are written as:

$$E_{x,0} = E_I cos(\omega_P t) \quad \text{(S3)}$$

$$E_{y,0} = E_I sin(\omega_P t) \quad \text{(S4)}$$

in which the ω_P is the rotating frequency. After propagating a distance of *z* along the graphene-coated *D*-shaped fiber, the components in *x*- and *y*- polarization become:

$$E_{x,z} = E_I cos(\omega_P t) exp(\alpha_x ct/n_x) \quad \text{(S5)}$$

$$E_{y,z} = E_I sin(\omega_P t) exp(\alpha_y ct/n_y) \quad \text{(S6)}$$

wherein the n_x and n_y are the indexes of the GDF in *x*- and *y*- polarizations; the α_x and α_y are the attenuation coefficients in *x*- and *y*- polarizations, respectively. The *c* is the light velocity in vacuum, and the L_G is the length of the graphene-coated *D*-shaped fiber. Hence, the total transmission power at point *z* is:

$$P_z = P_x + P_y = E_{x,z}{}^2 + E_{y,z}{}^2 = E_I{}^2 \left\{ \cos^2(\omega_P t) e^{2\alpha_x M_x} + sin^2(\omega_P t) e^{2\alpha_y M_y} \right\} \quad \text{(S7)}$$

wherein the M_x=$ct/n_x$and M_y=ct/n_y. Moreover, we take the polarization dependent saturable absorptions into consideration. The maximum power launched into the GDF fiber in our

experiment is ~ 3 W. Referring the effective field distributions calculated in S1.1, the power density in the GDF in our experiment was much lower than 1 GW/cm^2, which is far lower from the saturated limitation for a graphene based waveguide [S6]. Accordingly, we approximately regard the polarization dependent saturable absorption based transmission as [S7]:

$$P_{SX} = A_x e^{B_x P_x} \tag{S8}$$

$$P_{SY} = A_y e^{B_y P_y} \tag{S9}$$

Here, $A_x(u_c,L_G)$,$A_y(u_c,L_G)$and $B_x(u_c,L_G)$, $B_x(u_c,L_G)$are constants determined by the length and Fermi-level of graphene, in which u_c is the chemical potential of the graphene while L_G is the length of the GDF.

In practice, the difference between the M_x and the M_y is negligible. Applying the *Matlab*, and adopting parameters ω_P = 2×10^{5}π (100 kHz), 10^{6}π (500 kHz) and 2×10^{6}π π (1 MHz), the pulse series P_z(t) is shown in Fig. S1 (b). These results are also corresponding to the Fig. 4 in the text. The values of the α_x and α_y are from the measured results in Ref. [S8]. Moreover, when fixing the repetition rate at 100 kHz, by adopting different A_xA_y and B_xB_y, the pulsewidth is tunable, as shown in Fig. S1 (c). Fig. S1 indicates, by either increasing the saturable absorption ratio or the repetition rate, the pulses can be narrowed.

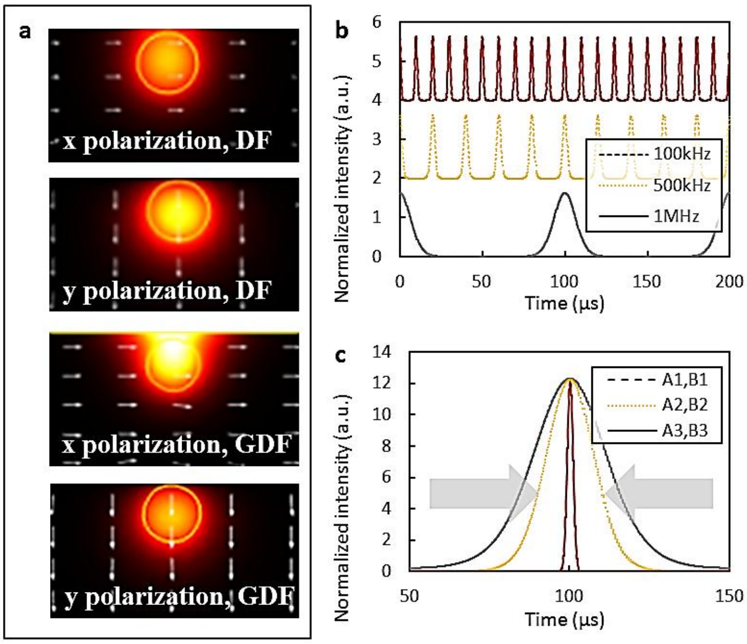

Figure S1 | Numerical simulation results. (a) Electric field distributions for the *D*-shaped

fiber (*DF*) and the graphene-coated *D*-shaped fiber (*GDF*). (b) Pulses generated by tuning the rotating speed at 100 kHz, 500 kHz, and 1MHz, with a normalized power. (c) Width of a single pulse, by adopting different parameters, the FWHMs could be 25 μs (grey), 12 μs (yellow), and 2 μs (red).

S2. Fabrication of the GDF.

GDF samples were fabricated by using the following steps shown in Fig. S2. Firstly, a soft and flexible PMMA/graphene was prepared; secondly, a specially designed *D*-shaped fiber was fabricated; thirdly, the PMMA/graphene hybrid was covered on the polished surface of the *D*-shaped fiber, finally, the PMMA film was removed. In the CVD process, graphene films were grown on Cu foils (Alfa Aesar, No. 13382), under 1000 ℃, 50sccm CH_4 and 50sccm H_2 [S9]. A thin PMMA film was spin-coated on the surface of graphene/Cu foil, to form a PMMA/graphene/Cu sandwich like structure. The thickness of the PMMA was ~1μm. Then the underlying Cu foil was chemically etched by 1M $FeCl_3$ solution, the obtained PMMA/graphene film is of good flexibility. On the other hand, by using mechanical polishing method in wet environment, the side of a single mode fiber (SMF-28e, Corning Inc.) was polished into a *D*-shaped fiber. The polishing process was repeated carefully to reduce the scattering loss [S10]. Moreover, to ensure the interactions between the transmitting light and the graphene film, the polishing depth was accurately controlled to be ~58μm (±0.5μm), which means the distance between the core to the polished surface is less than ~0.5μm. Subsequently, the flexible PMMA/graphene film was washed in DI water 3 times and transferred onto the side surface of the *D*-shaped fiber, which had been ultrasonically cleaned in sequence by acetone, ethanol and DI water. Then the PMMA/graphene/D-shaped fiber was dried at room temperature for 12 hours and baked at 180 ℃ for 10 min. Finally, the PMMA was removed by acetone vapor.

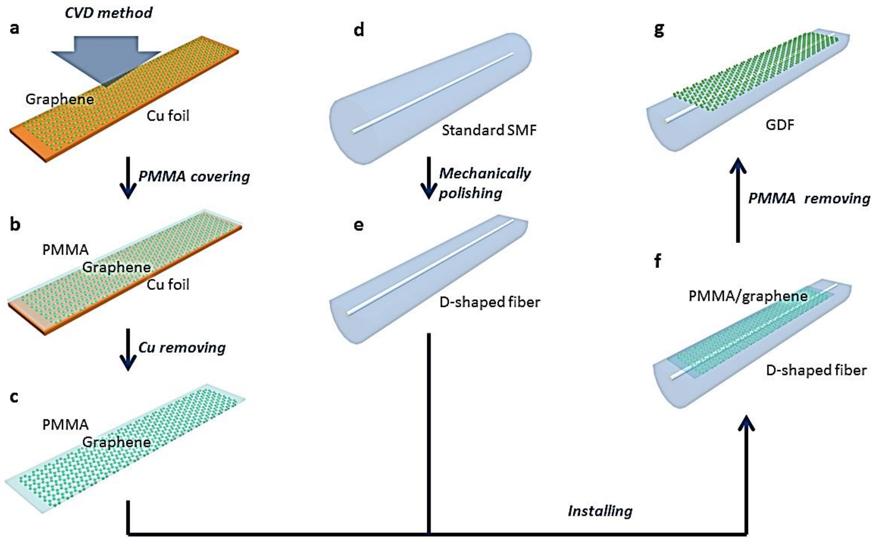

Figure S2 | Fabrication process of the GDF. a, By using the CVD method, a monolayer graphene was grown on a Cu foil. **b,** PMMA/graphene/Cu foil sandwich structure. **c,** PMMA/graphene flexible film. **d,** Standard SMF with core diameter 8μm and cladding diameter 125μm. **e**, By using mechanical polishing method, the standard SMF was fabricated to be a *D*-shaped fiber. **f**, Transfer of the PMMA/graphene onto the *D*-shaped fiber. **g,** PMMA removal.

S3. Extended characterization of the GDF.

Optical microscopes (OPM), scanning electronic microscopes (SEM) and Raman spectra were used to monitoring the fabrication process and the quality of the GDF sample, as shown in Fig. S3. Fig. S3(a) ~Fig. S3(e) show the OPM pictures for a typical as-fabricated GDF, with focusing on the boundary of the graphene coverage, with a series of amplification ratios. The OPM pictures demonstrate the combination of the GDF. Fig. S3(f) displays a SEM pictures of the sectional view and the uniform polished surface of the *D*-shaped fiber. The surface is smooth and uniform, and the polished depth is ~58 μm, i.e. ~0.5 μm away from the fiber core. Fig. S3(g) shows the Raman spectra of the graphene deposited on the *D*-shaped fiber, measured at 3 random positions, verifying that the graphene is of monolayer and high quality [S11-S13].

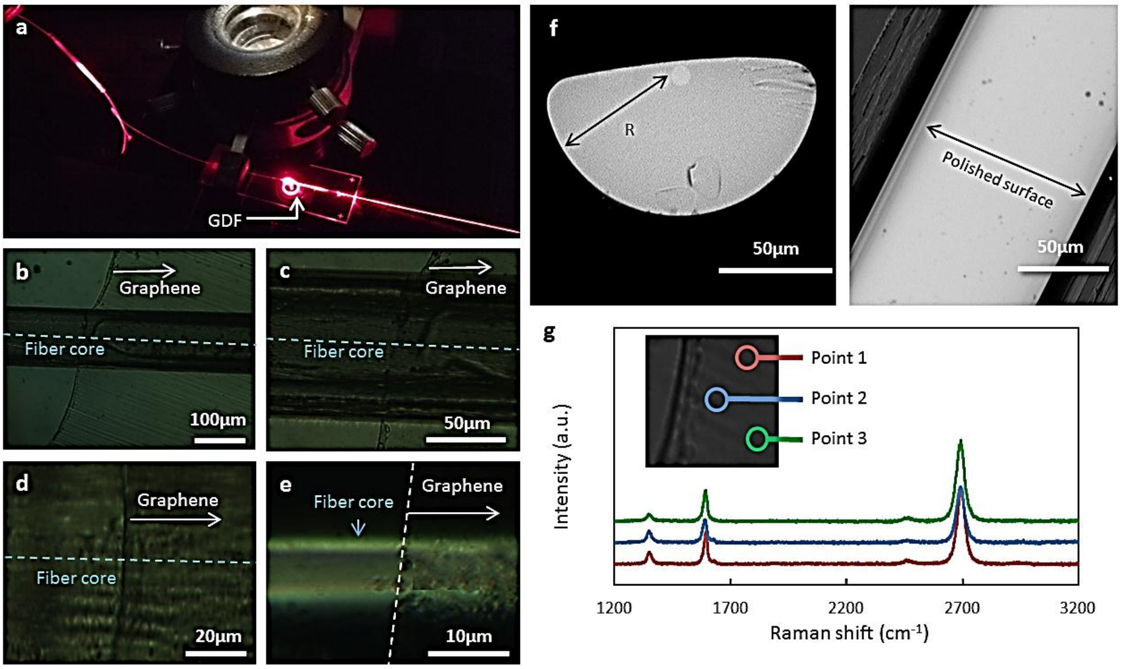

Figure S3 | Characterizations the GDF. a, A GDF with length of 16.7mm guiding 633nm light is illustrated under an optical microscope. **b** to**e**, Optical microscope images of the GDF. Here the bars in (**b**), (**c**), (**d**) and (**e**) are 100μm, 50μm, 20μm, and 10μm respectively. **f**, Scanning electron microscope image of the sectional view and the polished side of the *D*-shaped fiber. Here the scale bar is 50μm. **g,** SEM Raman spectra of graphene deposited on the *D*-shaped fiber measured from 3 different positions on the fiber, illustrating uniformity.

S4. Measurement setup.

Devices and instruments arranged in the experimental system (briefly shown in Fig. 1 in text) are specifically described in Fig. S4. A CW laser at 1455 nm (KEOPSYS, France) was adopted as the pump, whose maximum output power could be tunable up to 10 W. The FBG written by a 248nm laser with reflectivity of 0.95 was used to decrease the pump threshold for lasing, its Bragg peak width is 0.5 nm. The polarization rotator (*PR*) includes: an attenuator (ATT), a polarizer, a polarization modulator (PM, General Photonics, USA), and an Erbium-doped fiber amplifier (EDFA). The speed of the PM is tunable from 1 kHz to 3 MHz. The launched power in front of the graphene-coated *D*-shaped fiber is tunable up to 3.5 W. Finally, the output light was measured by using an optical spectrum analyzer (OSA) (Ando, Japan), an oscilloscope (Tektronix, USA, maximum sampling rate 5 GHz) and a

polarization analyzer (Agilent, USA)

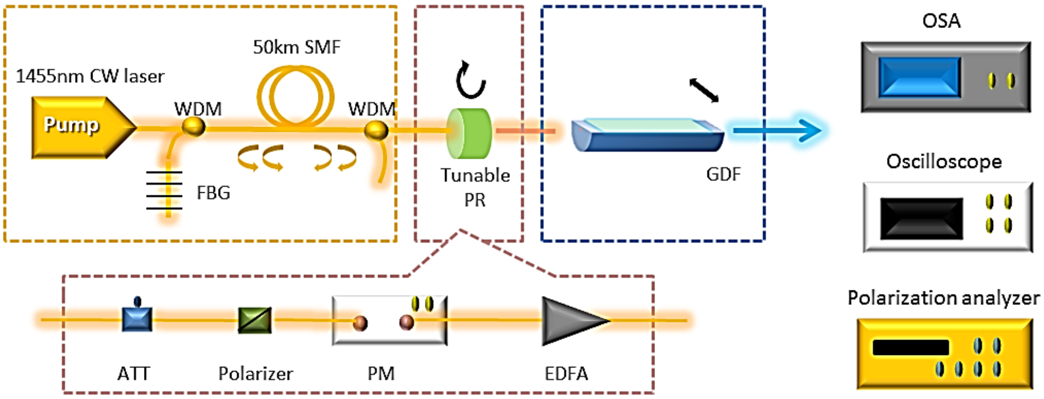

Figure S4 | Measurement setup. Here the CW random fiber laser, PR, and GDF are marked by yellow box, pink box, and blue box respectively.

S5. Measurement and simulation of the polarization dependent saturable absorption.

Saturable absorptions exist in the GDF. We measured the saturable absorption of the GDF, by using the experimental setup shown in Fig. S5(a). Here a polarization controller (PC) is used to adjust and fix the input polarization. The power launched into the graphene-coated *D*-shaped fiber can reach 3.5 W. The power meter with resolution of 0.1 dBm was used to detect the output power at 1550 nm. The transmittance could be calculated as P_B/P_A. Fig. S5(b) shows the transmissions of the *x*- and *y*- polarization measured by the power meter, *via* Channel *B*.

In addition, in this work, limited by the thermal damage threshold of the GDF, results under higher power (>3 W) are difficult to be provided. By using the formula $T = 1-\Delta T\times exp(-P/P_{sat})-T_{ns}$ [S14], we can approximately calculate the saturable absorption curve completely, as shown in Fig. S5(c). Here ΔT is the modulation depth, P is the input power, P_{sat} is the saturating power, and T_{ns} is the non-saturable absorbance. Considering the Fermi-level tenability of graphene, it is also supposed that under an ultrahigh power, the transmission of *y*-polarization would be higher than the *x*-polarization [S15-S16].

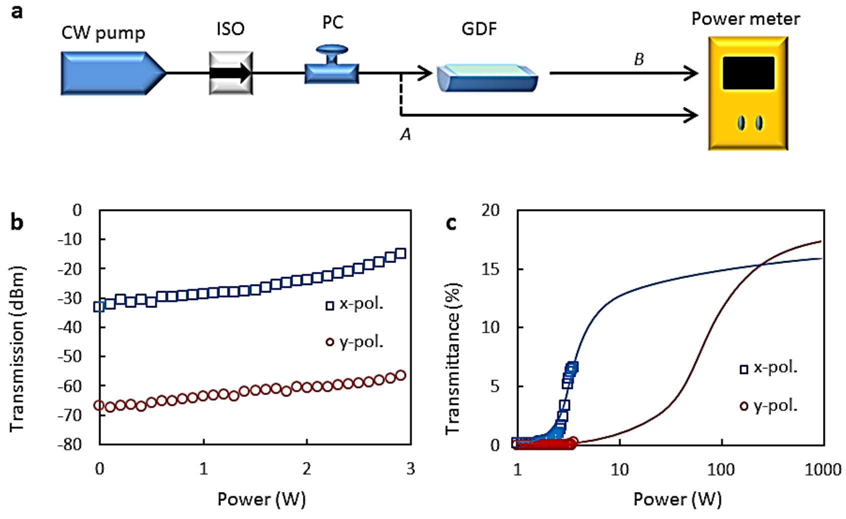

Figure S5 | Saturable absorption measurement of the GDF. a, Powers are measured before and after the GDF, so that the transmittance could be calculated precisely. **b,** Measured transmissions of the of the *x*- (blue boxes) and *y*- (red circles) polarization. **c,** Numerically calculated correlation of the power and transmittance of the GDF.

S6. GDF based polarization selectivity measurement.

According to its surface propagation properties, it has been verified that graphene could dramatically enhance the surface evanescent fields (see Fig. 1 in text) and induce a polarization dependent loss (see Fig. 2 in text) [S17-S20]. To verify the broadband polarization selectivity of the GDF, an experimental setup was built as shown in Fig. S6(a). Light from 1510 nm to ~ 1570 nm was launched from a tunable fiber laser (81960A, Agilent, USA), and measured by a high resolution OSA (8163B, Agilent, USA) and a power meter. The output power of the laser is fixed at 9.8 mW. A polarizer was used to control the launched polarizations. The measured spectra are shown in Fig. 2(a) in text.

Moreover, Poincaré representation of the graphene pulsed random fiber laser measurements are shown in Fig. 6(b), before the PR (Point A), after the PR (Point B), and after the GDF (Point C). Modulated by the PR and the GDF, the CW random fiber lasing is tuned to highly-polarized pulses.

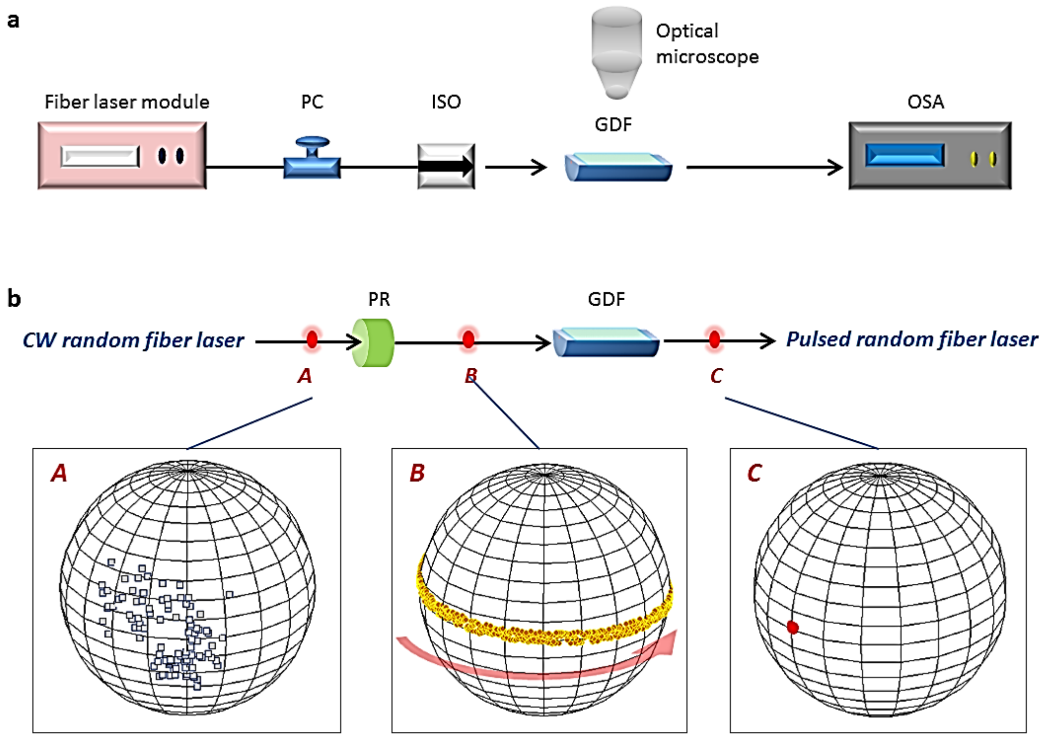

Figure S6 | GDF works as a polarization selector. a, Experimental setup to measure the polarization selectivity. **b,** Test points: before the PR (Point A), after the PR (Point B), and after the GDF (Point C).

S7. Spectra of the pulsed random fiber laser.

The nonlinear pulse reshaping of the pulses could also be verified by observing the spectral changes, considering the Fourier transformations [S21-S22]:

$$P_T\left(j\Omega\right)=\int_0^{+\infty}P_T\left(t\right)e^{-j\Omega}dt \qquad \text{(S10)}$$

With increasing the power launched into the GDF, more complicated nonlinear effects appear, such as self-phase-modulations and chirps, which contribute the spectral broadening. Fig. S7(a) and S7(b) concludes the correlation of “Power vs 3dB-width” and “Power vs 30dB-width” for the graphene pulsed random fiber laser.

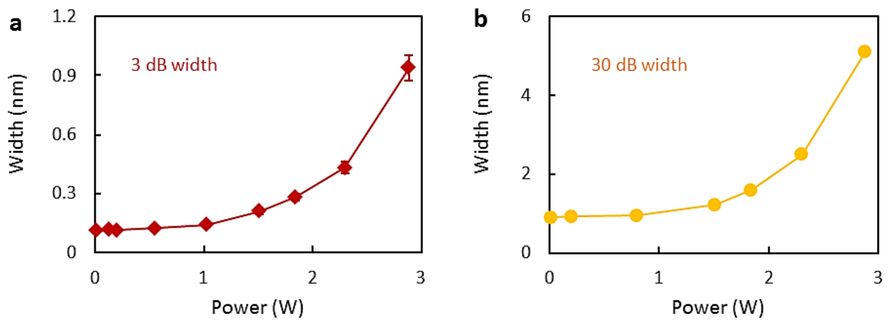

Figure S7 | Spectral broadening of the graphene pulsed random fiber laser. a, Power vs 3dB-width. **b,** Power vs 30dB-width.

Supplementary References

S1. B. C. Yao, Y. Wu, A. Q. Zhang, Y. J. Rao, Z. G. Wang, Y. Cheng, Y. Gong, W. L. Zhang, Y. F. Chen, Y. R. Li, and K. S. Chiang, Graphene enhanced evanescent field in microfiber multimode interferometer for highly sensitive gas sensing, Opt. Express **22**, 28155-28162 (2014).

S2. A. W. Synder and J. D. Love, Optical Waveguide Theory, *Chapman and Hall*, 1983).

S3. C. J. Docherty, C. T. Lin, H. J. Joyce, R. J. Nicholas, L. M. Herz, L. J. Li, and M. B. Johnston, Extreme sensitivity of graphene photoconductivity to environmental gases, Nat. Comm. **3**, 2235 (2012).

S4. Y. Wu, B.C. Yao, A.Q. Zhang, X.L. Cao, Z.G. Wang, Y.J. Rao, Y. Gong, W.L. Zhang, Y.F. Chen, and K.S. Chiang, Graphene-based D-shaped fiber multicore mode interferometer for chemical gas sensing, Opt. Lett. **39**, 6030-6033 (2014).

S5. B.C. Yao, Y. Wu, Z.G. Wang, Y. Cheng, Y.J. Rao, Y. Gong, Y.F. Chen, and Y.R. Li, Demonstration of complex refractive index of graphene waveguide by microfiber-based Mach–Zehnder interferometer, Opt. Express **21**, 29818-29826 (2013).

S6. W. Li, B. Chen, C. Meng, W. Fang, Y. Xiao, X. Li, Z. Hu, Y. Xu, L. Tong, H. Wang, W. Liu, J. Bao, and Y. Shen, Ultrafast all-optical graphene modulator, Nano. Lett. **14**, 955−959 (2014).

S7. H. Haus, Theory of mode locking with a slow saturable absorber, IEEE J Quantum Electron. **11**, 736-746 (1975).

S8. Q. Bao, H. Zhang, B. Wang, Z. Ni, C. H. Y. X. Lim, Y. Wang, D. Y. Tang, and K. P. Loh,

Broadband graphene polarizer, Nat. Photonics **5**, 411–415 (2011).

S9. Z. G. Wang, Y. F. Chen, P. J. Li, X. Hao, J. B. Liu, R. Huang, and Y. R. Li, Flexible graphene-based electroluminescent devices, ACS Nano **5**, 7149–7154 (2011).

S10. Y. Wu, B. C. Yao, A. Q. Zhang, X. L. Cao, Z. G. Wang, Y. J. Rao, Y. Gong, W. Zhang, Y. F. Chen, and K. S. Chiang, Graphene based D-shaped fiber multi-core modes interferometer for chemical gas sensing, Opt. Lett. **39**, 6030-6033 (2014).

S11. A. C. Ferrari, J. C. Meyer, V. Scardaci, C. Casiraghi, M. Lazzeri, F. Mauri, S. Piscanec, D. Jiang, K. S. Novoselov, S. Roth, and A. K. Geim, Raman spectrum of graphene and graphene Layers, Phys. Rev. Lett. **97**, 187401 (2006).

S12. Y. K. Koh, M. H. Bae, D. G. Cahill, and E. Pop, Reliably counting atomic planes of few-layer graphene, ACS Nano **5**, 269–274 (2011).

S13. A. Reina, X. T. Jia, J. Ho, D. Nezich, H. Son, V. Bulovic, M. S. Dresselhaus, and J. Kong, Large area, few-layer graphene films on arbitrary substrates by chemical vapor deposition, Nano Lett. **9**, 30–35 (2009).

S14. J. Du, Q. Wang, G. Jiang, C. Xu, C. Zhao, Y. Xiang, Y. Chen, S. Wen and H. Zhang, Ytterbium-doped fiber laser passively mode locked by few-layer Molybdenum Disulfide (MoS_2) saturable absorber functioned with evanescent field interaction, Scientific Report **4**, 03149 (2014).

S15. A. Grigorenko, M. Polini, and K. Novoselov, Graphene plasmonics, Nat. Photon.**6,** 749–758 (2012).

S16. M. Liu, X. Yin, E. Avila, B. Geng, T. Zentgraf, L. Ju, F. Wang, and X. Zhang, A graphene-based broadband optical modulator, Nature **474**, 64-67 (2011).

S17. B. C. Yao, Y. Wu, A. Q. Zhang, Y. J. Rao, Z. G. Wang, Y. Cheng, Y. Gong, W. L. Zhang, Y. F. Chen, Y. R. Li, and K. S. Chiang, Graphene enhanced evanescent field in microfiber multimode interferometer for highly sensitive gas sensing, Opt. Express **22**, 28155-28162 (2014).

S18. S. A. Mikhailov and K. Ziegler, New electromagnetic mode in graphene, Phys. Rev. Lett. **99**, 016803 (2007).

S19. E. H. Hwang and S. D. Sarma, Dielectric function, screening, and plasmons in two-dimensional grapheme, Phys. Rev. B **75**, 205418 (2007).

S20. Y. Wu, B. Yao, Y. Cheng, X. Liu, Y. Gong, and Y. Rao, Hybrid graphene-microfiber waveguide for chemical gas sensing, J. Sel. Top. Quantum Electron. **20**, 4400206 (2014).

S21. A. Oppenheim, A. Willsky, and S. Nawab, *Signals and systems*, (Prentice Hall, 2002).

S22. Q. Bao, H. Zhang, Z. Ni, Y. Wang, L. Polavarapu, Z. Shen, Q. Xu, D. Tang, and K. P. Loh, Monolayer graphene as a saturable absorber in a mode-locked laser, Nano Res. **4**, 297-307 (2011).
